# Supplementary material for: Mapping phenotypic heterogeneity in melanoma onto the epithelial-hybrid-mesenchymal axis
Source: Front Oncol. 2022 Aug 8;12:913803. doi: 10.3389/fonc.2022.913803 (PMC9395132; doi:10.3389/fonc.2022.913803)
Supplement: Supplementary file 1 [file Presentation_1.pdf]

Supplementary Figures

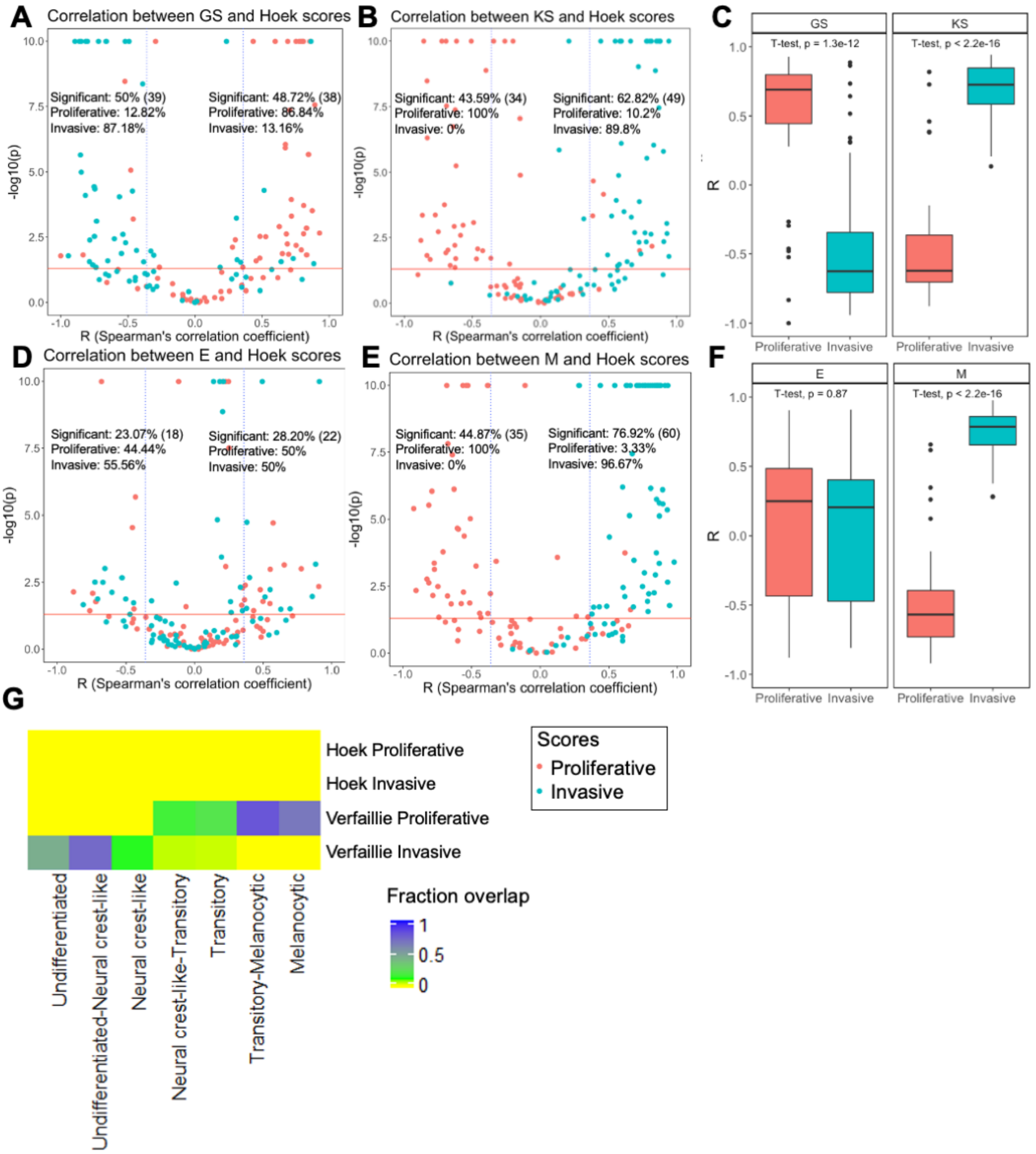

Fig S1 *Volcano plots depicting the Spearman's correlation coefficient and -log₁₀(p-value) for Hoek proliferative and invasive gene set and* ***A.*** *76GS EMT scoring metric* ***B.*** *KS EMT scoring metric* ***C.*** *Boxplot depicting range of correlation coefficients for KS and GS* ***D.*** *Epithelial gene set* ***E.*** *Mesenchymal gene set.* ***F.*** *Boxplot depicting range of correlation coefficients for E and M scores Inset labelled "Significant" is calculated as 100* number of significant points under specified cut-off /78. Absolute number of significant points for the specified cut of is mentioned in brackets. "Proliferative" and "Invasive" labels represent the percentage of significant correlations that are between the EMT score and proliferative score or invasive score, respectively.* ***G.*** *Extent of overlap between Verfaillie, Hoek gene sets (Proliferative and invasive phenotype gene sets) and Tsoi gene sets (intermediate phenotype gene sets)*

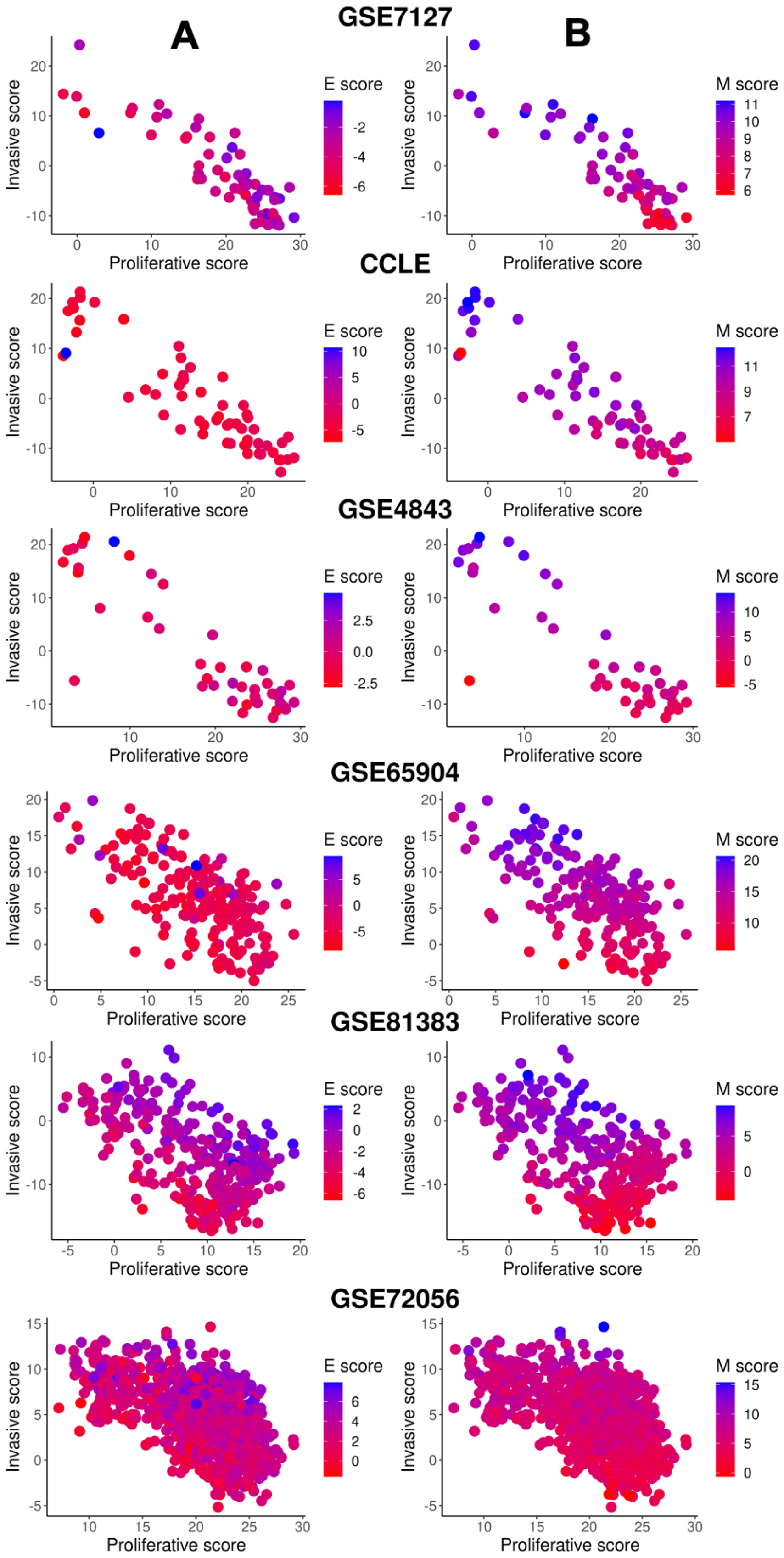

Fig S2 A. *Two dimensional EMT plots depicting the variation of epithelial score gradient along the proliferative-invasive score axes.* ***B.*** *Two dimensional EMT plots depicting the variation of mesenchymal score gradient along the proliferative-invasive score axes.*

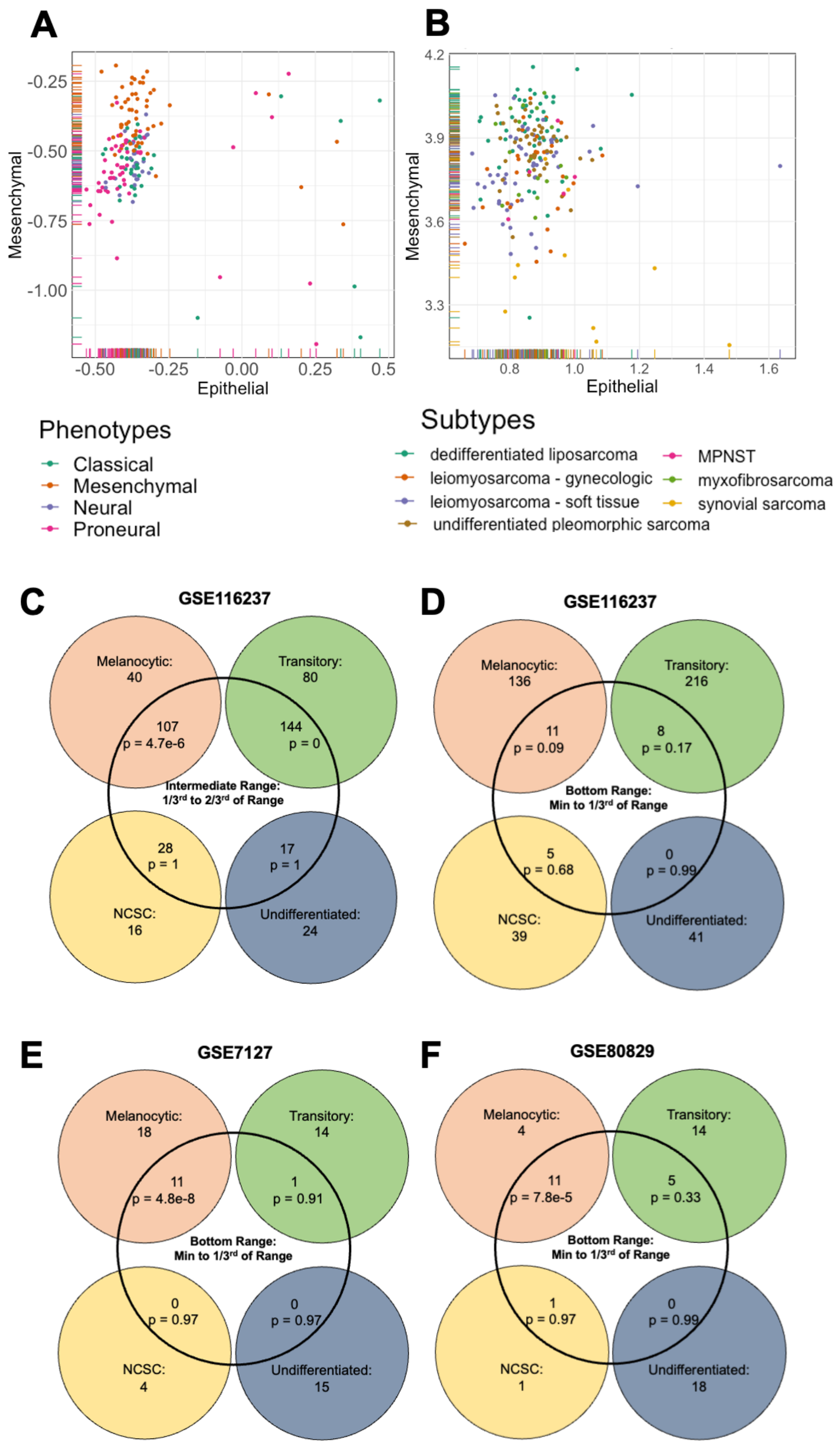

***Fig S3** 2D EMT plots with phenotypes for **A.** TCGA GBM and **B.** TCGA SARC datasets. Each dot denotes a TCGA sample and its corresponding epithelial and mesenchymal scores are plotted. Venn diagram depicting **C.** The intersection of the four phenotype scores of samples and intermediate M scores in GSE116237. p represents p-value for the conditional probability that a sample belongs to the phenotype given that they lie in the intermediate M score range**.** The intersection of the four phenotype scores of samples and bottom M scores in **D.** GSE116237 **E.** GSE7127 **F.**GSE80829. p represents p-value for the conditional probability that a sample belongs to the phenotype given that they lie in the bottom M score range.*

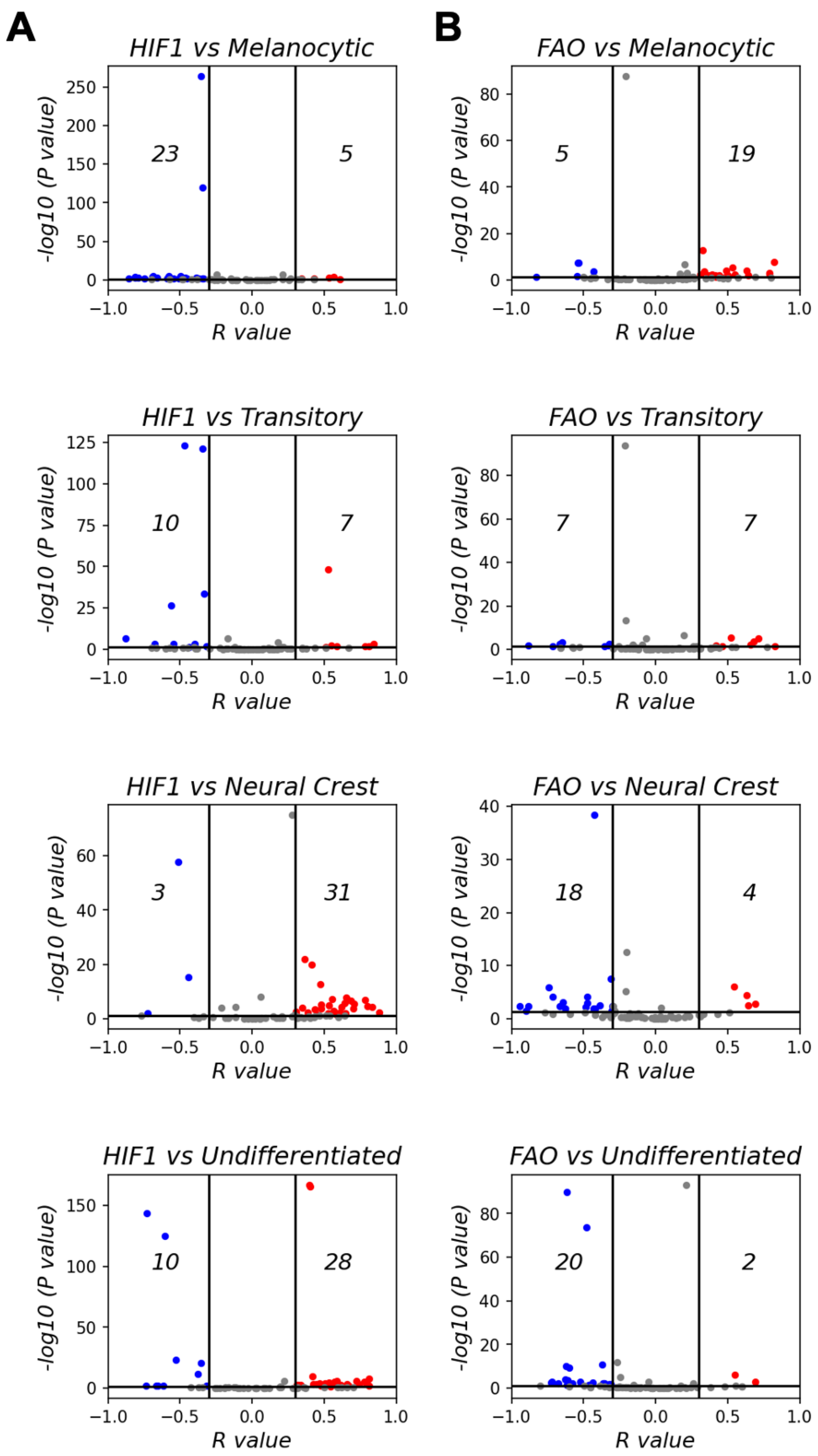

Fig S4 *Volcano plots depicting the Spearman's correlation coefficient and -log*$_{10}$*(p-value) for* ***A.*** *HIF1 signature and Tsoi gene set.* ***B.*** *Fatty acid oxidation (FAO) gene set and Tsoi gene set.*

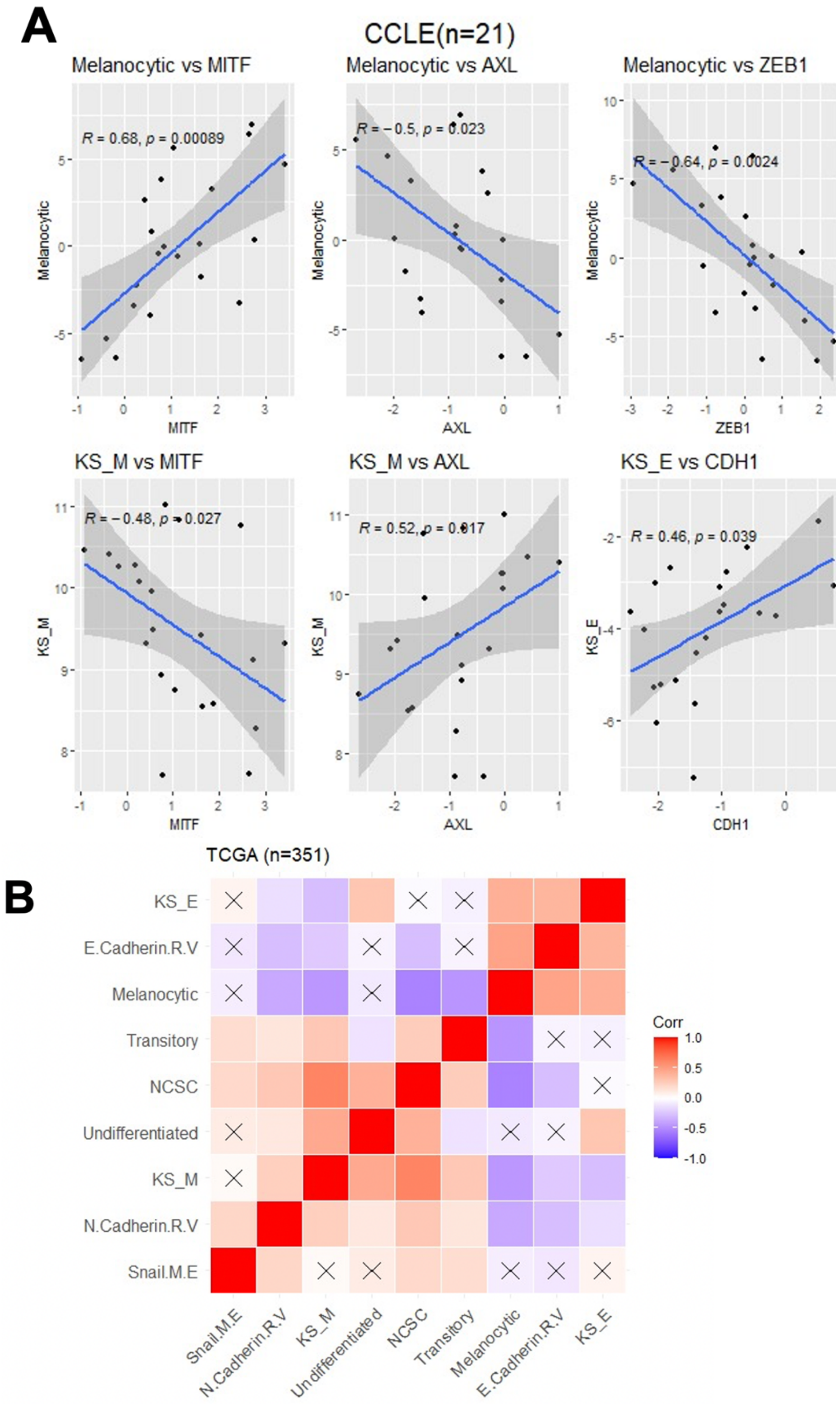

***Fig S5: A.** Scatter plot for CCLE (n=21) depicting correlation between transcriptomic signatures (Melanocytic, KS mesenchymal score (KS_M) and KS Epithelial score (KS_E) and proteomic marker levels (MITF, AXL, CDH1, ZEB1). R indicates correlation coefficient. **B.** Correlation matrix for TCGA (n=351) for various transcriptomic signatures (melanocytic, Transitory, NCSC, Undifferentiated, KS_M, KS_E) and proteomic marker levels (Snail, N-cadherin, E-cadherin). Color bar indicates correlation coefficient. Crosses indicate p-value <0.05 for a given pair-wise correlation.*
